# Laminin-Derived Peptides and Isoforms: A Multifaceted Review of Their Roles in Cancer, Neurodegeneration, Inflammation, Tissue Regeneration, and Exercise Physiology

**DOI:** 10.3390/cimb48101040

**Published:** 2026-10-08

**Authors:** Elcirley Luz Silva, Gilberto Kocerginsky, Cláudia Marlise Balbinotti Andrade, Romeu Paulo Martins Silva, Aníbal Monteiro de Magalhães-Neto, Luis Carlos Oliveira Gonçalves

**Affiliations:** 1 World ParajiuJitsu Federation, Wilmington, DE 19805, USA; contact@wpjjf.com; 2 Gilberto Kocerginsky Institute of Medicine and Health, Curitiba 80430-180, Paraná, Brazil; gkwellness@hotmail.com; 3 Department of Basic Health Sciences, Faculty of Medicine, Cuiabá Campus, Federal University of Mato Grosso, Cuiabá 78060-900, Mato Grosso, Brazil; claudia.andrade@ufmt.br; 4 Center for Health Sciences, Postgraduate Program in Nutrition and Health, Maruípe Campus, Federal University of Espírito Santo, Vitória 29075-910, Espírito Santo, Brazil; romeu.silva@ufes.br; 5 Biologial and Health Science Institute, Araguaia Campus, Federal University of Mato Grosso, Pontal do Araguaia 78698-000, Mato Grosso, Brazil; anibal.neto@ufmt.br; 6 Institute of Biotechnology, Laboratory of Biochemistry and Molecular Biology (LABIBI), Umuarama Campus, Federal University of Uberlândia, Uberlândia 38408-100, Minas Gerais, Brazil

**Keywords:** YIGSR, IKVAV, C16, AG73, laminin-111, laminin-332, laminin-511, laminin γ2, polylaminin

## Abstract

Background: Laminins are heterotrimeric basement membrane glycoproteins whose chain composition, molecular processing, receptor engagement, and cellular source determine their biological effects. Methods: We conducted an integrative narrative search of PubMed and manually screened reference lists for studies of laminin isoforms, laminin-derived peptides, and polylaminin in cancer, neurological disorders, inflammation, tissue regeneration, wound healing, and exercise-related adaptation. Results: The evidence is heterogeneous and is predominantly mechanistic, in vitro, or preclinical. Laminin isoforms and derived peptides can produce opposing effects depending on the isoform or sequence, receptor, presentation format, tissue context, disease stage, and experimental model. Polylaminin is an experimental biomaterial with encouraging but preparation-dependent preclinical activity in selected neural repair models and in vitro stem cell culture. Conclusions: Laminin-based interventions should be developed in an indication-specific manner and require mechanistic validation, controlled delivery, dose–response characterization, safety assessment, reproducible manufacturing, and clinical validation before therapeutic translation.

## 1. Introduction

Laminins are heterotrimeric glycoproteins assembled from five α chains (α1–α5), three β chains (β1–β3), and three γ chains (γ1–γ3). The three-number laminin nomenclature identifies the α, β, and γ chains in that order; for example, laminin-111 contains α1, β1, and γ1, whereas laminin-332 contains α3, β3, and γ2. Reported heterotrimers differ in tissue distribution, receptor usage, matrix assembly, and biological function, and theoretical chain combinations should not be treated as equally abundant or equally characterized. Laminin-derived peptides, including YIGSR from laminin β1 and IKVAV from laminin α1, reproduce selected domain activities but should not be assumed to reproduce the functions of the intact parent heterotrimer [1,2,3,4,5,6,7,8,9,10].

The basement membrane is a specialized extracellular matrix composed principally of laminins, type IV collagen, nidogens, and proteoglycans such as perlecan. Laminins interact with cell-surface receptors, including integrins, dystroglycan, syndecans, Lutheran/BCAM, and the 37/67-kDa laminin receptor. These interactions regulate adhesion, matrix organization, migration, survival, differentiation, and barrier function. Receptor effects are isoform- and domain-dependent; downstream pathways such as PI3K/AKT, FAK/Src, MAPK/ERK, or STAT3 should be described only with the relevant laminin ligand, receptor, experimental model, and cellular outcome. Perlecan should be considered a basement-membrane proteoglycan and matrix organizer rather than a cell-surface laminin receptor [11,12,13,14,15,16,17,18].

**Figure 1:**
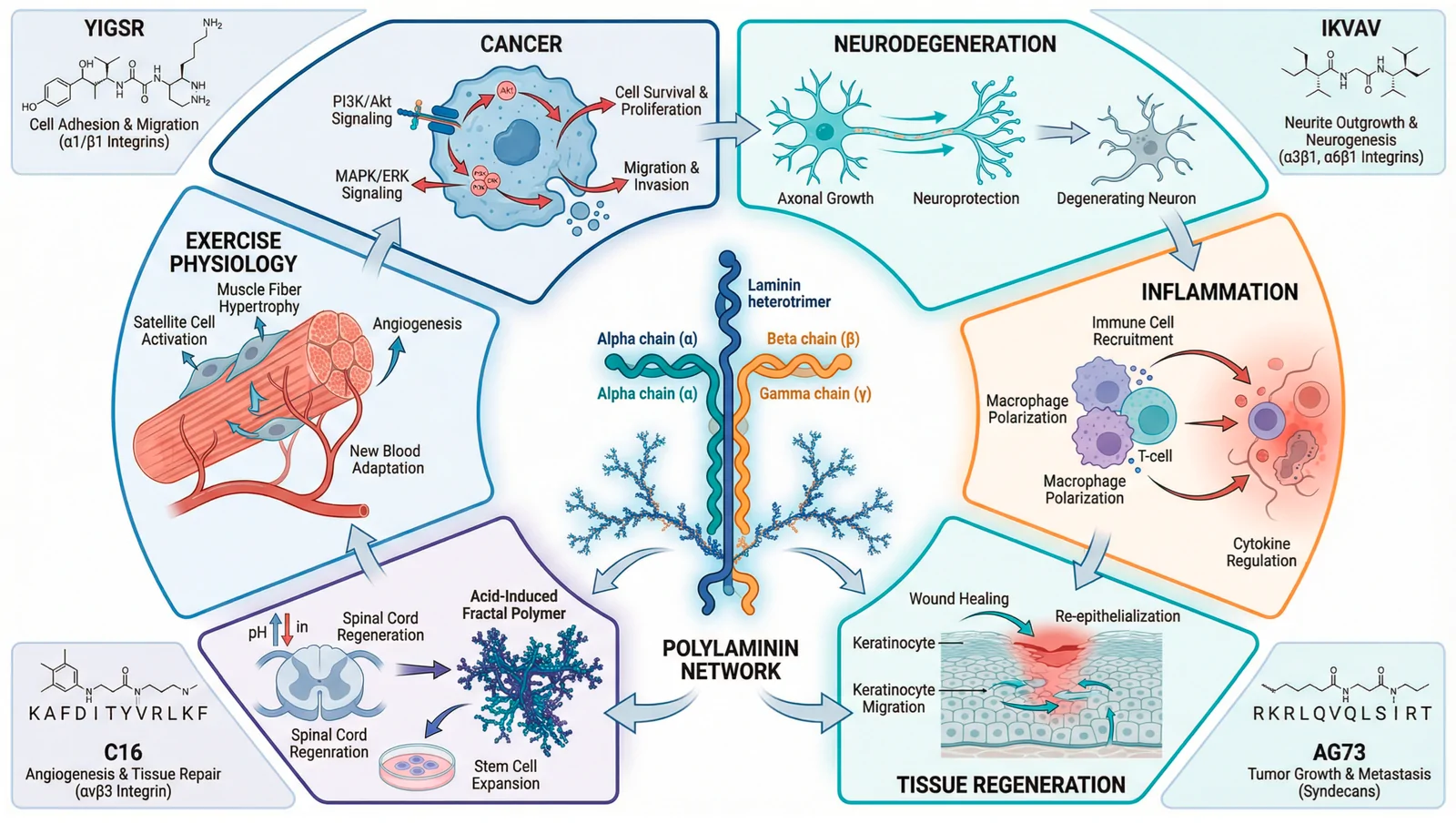
Integrative overview of laminin heterotrimers, laminin-derived peptides, and polylaminin across cancer, neurodegeneration, inflammation, tissue regeneration, exercise physiology, and spinal cord repair.

This integrative narrative review examines laminin isoforms, laminin-derived peptides, and polylaminin across cancer, neurological disorders, inflammation, tissue regeneration, wound healing, and exercise-related adaptation. We organize the evidence according to laminin chain or peptide sequence, receptor or binding partner, producing cellular compartment, experimental model, biological outcome, and evidence level. We also identify the limitations that currently prevent generalization to clinical therapy, including incomplete mechanistic validation, preparation-dependent activity, delivery constraints, immunogenicity, manufacturing requirements, and the scarcity of controlled human studies. Figure 1 summarizes the laminin entities and biological contexts discussed in this review.

## 2. Materials and Methods

We conducted an integrative narrative search of PubMed using combinations of terms related to laminin, laminin-derived peptides, laminin isoforms, cancer, neurodegeneration, inflammation, tissue regeneration, wound healing, exercise physiology, physical activity, and muscle adaptation. PubMed was selected as the primary database because the objective was to map the laminin-specific mechanistic and translational literature within an integrative narrative framework. We did not search Embase, Scopus, or Web of Science; this limitation may have omitted records indexed exclusively in those databases. The search was limited to English-language records and included no date restriction. We manually screened the reference lists of retrieved articles for additional relevant studies. We considered mechanistic, in vitro, ex vivo, animal, observational human, and interventional human studies when available. We did not apply a formal PRISMA, SANRA, JBI, or other validated risk-of-bias instrument. Instead, we qualitatively appraised studies by study design, experimental model, laminin or peptide identity, perturbation, comparator, endpoint, and evidence level. Because the available records do not permit reconstruction of a database-wide systematic search, formal screening counts, independent reviewer procedures, or quantitative risk-of-bias scoring, this article is presented as an integrative narrative review rather than a systematic review or meta-analysis.

## 3. Laminin Overview

### 3.1. Structural Organization and Biological Functions of Laminins

Laminins are cross-shaped heterotrimers with a molecular mass of approximately 400–900 kDa, depending on the isoform. The α, β, and γ chains contain distinct N-terminal and C-terminal domains that contribute to heterotrimer assembly, matrix polymerization, receptor binding, and proteolytic processing. The α-chain LG domains contain important cell-binding sites, including the IKVAV sequence in laminin α1; YIGSR is a laminin β1-derived sequence and should not be generically assigned to the α-chain LG domain. The β and γ chains also contribute to receptor interactions and bioactive fragments. Laminin polymerization and basement membrane organization involve short-arm interactions and receptor-assisted anchorage, and should be distinguished from the acid-induced cell-free polymerization used to produce polylaminin [19,20,21,22,23,24,25,26,27,28].

The basement membrane provides structural support, selective barrier function, and a platform for receptor-mediated signaling. Laminins can bind or present growth factors and cytokines, and proteolytic remodeling can both release matrix-associated factors and generate laminin fragments with independent signaling activity. These effects are protease-, isoform-, tissue-, and context-dependent. The loss of an intact laminin network and the gain of a bioactive laminin fragment should therefore be analyzed as distinct biological events [29,30,31,32,33,34,35,36]. Table 1 summarizes representative laminin heterotrimers and their evidence levels.

### 3.2. Laminins in Cancer: Expression, Signaling, and Therapeutic Implications

Laminin biology in cancer is isoform-, compartment-, processing-, and stage-dependent. An intact basement membrane may restrain invasion, whereas tumor-associated laminin deposition, receptor engagement, proteolytic processing, or loss of matrix anchorage may promote invasion and survival. Therefore, altered laminin expression or poor prognosis should not be interpreted as proof of a causal tumor-promoting function. Each claim should identify the laminin isoform, producing cell, molecular form, receptor or partner, tumor type, perturbation, and endpoint [37,38,39,40,41,42,43,44,45,46].

Laminin-332 can support epithelial adhesion and wound repair but may promote migration and invasion in tumor-associated contexts through α3β1, α6β4, syndecan-1, EGFR, and downstream cytoskeletal or survival signaling. The γ2 chain is particularly sensitive to proteolytic processing; full-length and processed γ2 forms should not be treated as biologically equivalent. Laminin-511/LM-α5 should likewise be interpreted by the source. In colorectal liver metastasis, inflammatory myeloid-cell-conditioned signals induced tumor-cell LAMA5, and its knockdown reduced metastatic growth and vessel branching; this finding should not be generalized to all endothelial or stromal LM-511 [47,48,49,50,51,52,53,54,55,56].

Laminin-derived peptides have opposing, model-specific effects in cancer. YIGSR, a β1-derived sequence, inhibited melanoma invasion and experimental lung colonization in defined models. In contrast, IKVAV, AG73, and C16 have promoted angiogenesis, protease activity, invadopodia activity, invasion, or metastasis in other preclinical models. Free peptide, multimeric peptide, tethered peptide, scaffold-bound peptide, and conjugated peptide are not interchangeable preparations; the direction of effect must therefore be linked to sequence, presentation, receptor, dose, and model [57,58,59,60,61,62,63,64].

The activity of laminin-derived peptides cannot be compared across studies without considering receptor context, presentation format, concentration, and tissue microenvironment. The available literature does not provide a uniform set of binding affinities or effective concentration ranges for YIGSR, IKVAV, AG73, and C16. The same peptide may behave as a competitive antagonist, agonist-like ligand, adhesion cue, or scaffold-associated signal depending on whether it is free, multimerized, conjugated, tethered, or presented on a biomaterial. Integrin engagement, syndecan binding, matrix stiffness, protease activity, growthfactor availability, and the phenotype of the responding cell should therefore be interpreted as context-dependent determinants rather than as universal properties of the peptide sequence.

C16 is a laminin γ1-derived peptide with the sequence KAFDITYVRLKF; C16 is not a laminin α1-derived peptide. In defined models, C16 promoted endothelial adhesion, tumor-cell migration, MMP-9 secretion, invadopodia activity, or pulmonary metastasis, whereas a scrambled control did not reproduce the same activity. These findings do not support describing C16 as a general anti-cancer agent. Claims about apoptosis or tumor inhibition were retained only if the cited primary study directly tested the stated model, formulation, concentration, and endpoint [65,66,67,68].

IKVAV is a laminin α1-derived sequence with neuritogenic and adhesion-related activity. Its effects in cancer are context-dependent and should not be described as universally anti-tumor. Claims involving autophagy, CREB, BDNF, or NGF must identify the exact cancer model and primary experiment; otherwise they should be removed or presented as hypotheses. The peptide section should distinguish neural, angiogenic, wound-healing, and cancer evidence rather than combining them as a single therapeutic mechanism [69,70,71,72].

### 3.3. Laminins in Neurodegeneration, Neuroprotection, and Neuroregeneration

Laminin functions in the nervous system should be separated by endpoint. In cultured neurons, laminin can support survival and neurite extension through domain- and receptor-dependent mechanisms. β1-integrin-dependent laminin signaling has also been linked to axon formation and microtubule organization in developmental models. These endpoints are not equivalent to axonal guidance, synaptic integration, or functional recovery, and each claim should identify the laminin preparation, substrate, receptor, model, and outcome [73,74,75,76].

Consider neural and neuromuscular effects in distinct subsections. Neuronal laminin α5 has been associated with synapse stabilization, while laminin-521 supports defined human pluripotent stem cell cortical network cultures. Laminin receptors in Schwann cells, including α6β1, dystroglycan, and α6β4, have stage-specific roles in axon sorting and myelination. IKVAV- or laminin-containing substrates may support neural stem cell attachment, morphology, or neurite extension in vitro, but these findings do not establish in vivo neural stem cell differentiation or clinical regeneration [77,78,79,80].

For neurodegeneration, current evidence should be classified as association, mechanism, or intervention/rescue. Astrocytic or pericytic laminin loss can produce blood–brain barrier and pericyte abnormalities in genetic or conditional models, and MMP-9-mediated laminin proteolysis can contribute to neuronal apoptosis after acute focal ischemia. These findings support cell-source- and context-specific mechanisms but do not prove that laminin alterations initiate chronic Alzheimer’s disease, Parkinson’s disease, or ALS. Pharmacological rescue in familial Alzheimer’s fibroblasts should be identified as a non-neuronal in vitro model. Laminin-based neuroprotection should therefore be presented as a preclinical hypothesis rather than an established disease-modifying therapy [81,82]. Table 2 summarizes the evidence for laminin-related neural effects.

### 3.4. Laminins in Inflammation and Immune Regulation

Laminins can influence inflammation through two separable mechanisms. Direct regulation occurs when leukocytes or macrophages respond to a laminin ligand or peptide through a defined receptor. Indirect regulation occurs when laminin organization or proteolysis changes endothelial or epithelial barrier resistance, chemokine presentation, basement-membrane permissiveness, or exposure of cryptic fragments. The sequence of adhesion, migration, transendothelial passage, and tissue infiltration was characterized for the specific immune cell and tissue compartment examined [83,84,85,86].

Endothelial laminin-511 should not be described simply as a promoter of leukocyte recruitment. In a defined endothelial model, laminin-511 increased barrier resistance, stabilized VE-cadherin through β1/β3-integrin-associated RhoA signaling, reduced CD99L2, and inhibited neutrophil transmigration. The result is context-dependent and should not be generalized to all leukocyte subsets, tissues, or laminin isoforms [87,88,89].

YIGSR effects on macrophages should be described as concentration-dependent and model-specific. Lower and higher concentrations produced different inflammatory marker and secretome responses in the reported human and murine systems; this does not justify a universal statement that YIGSR suppresses NF-κB or converts macrophages to an M2 phenotype. AG73 should be discussed primarily as a syndecan-binding peptide with angiogenesis and morphogenesis evidence unless a directly matching immune-cell recruitment study is verified [90,91,92,93].

Claims concerning laminin-332 and regulatory T-cell differentiation require a specific primary source, cellular source, receptor, experimental system, and perturbation. If these details cannot be verified, replace the current therapeutic conclusion with “Laminin isoforms and laminin-derived peptides may influence inflammatory-cell behavior, but the direction and mechanism are context-dependent and remain predominantly preclinical [94,95].

### 3.5. Laminins in Tissue Regeneration and Wound Healing

Tissue regeneration and wound healing are complex biological processes that require coordinated interactions between multiple cell types and signaling pathways, and laminins play a critical role in orchestrating these processes [96]. Laminin-332 is essential for proper basement membrane formation in the skin and plays a critical role in wound healing through multiple mechanisms [97]. During the early stages of wound healing, laminin-332 is upregulated at the wound margin and promotes keratinocyte migration by activating integrin-mediated signaling [98]. α3β1 integrin binding to laminin-332 activates FAK and Src family kinases, which regulate focal adhesion dynamics and promote cell migration [99].

Laminin-511 also plays important roles in tissue regeneration and wound healing. Laminin-511 expression is upregulated during wound healing, and this isoform has been shown to promote keratinocyte migration and re-epithelialization [100]. Laminin-511 interacts with α6β1 integrin on keratinocytes, promoting their migration and differentiation [101]. Furthermore, laminin-511 has been shown to promote the differentiation of bone marrow-derived mesenchymal stem cells into osteoblasts, suggesting its role in bone regeneration [102].

Laminin-111 has been shown to enhance wound healing in animal models, particularly in diabetes-associated impaired wound healing [103]. Laminin-111 promotes keratinocyte migration and proliferation by activating integrin-mediated signaling pathways [104]. Additionally, laminin-111 has been shown to promote angiogenesis and accelerate new blood vessel formation during tissue healing [105]. These pro-angiogenic effects of laminin-111 are mediated by the activation of endothelial cell integrins and the upregulation of VEGF signaling [106].

Laminin-derived peptides have also been shown to promote tissue regeneration and wound healing. The AG73 peptide has been shown to promote angiogenesis and accelerate wound healing by promoting endothelial cell proliferation and tube formation [107]. AG73 also promotes the recruitment of immune cells involved in tissue repair and the production of growth factors that support tissue regeneration [108]. The YIGSR peptide has been shown to promote wound healing by promoting keratinocyte migration and differentiation [109]. Additionally, YIGSR can promote angiogenesis by promoting endothelial cell proliferation and tube formation [110].

### 3.6. Laminins and Exercise Physiology: Molecular Adaptations and Therapeutic Applications

Exercise-related laminin biology should be framed as a mechanistic interface between mechanical load, basal lamina remodeling, satellite cell behavior, neuromuscular junction organization, and vascular adaptation. Activation of muscle stem cells can remodel the local extracellular matrix and alter laminin α1/α5 deposition; genetic or receptor perturbations in injury models support a role for laminin-rich niches in satellite cell expansion and regeneration. These findings do not prove that exercise increases a specific laminin isoform in humans, improves athletic performance, or produces a therapeutic effect. Neuromuscular junction laminins, including α4, α5, and β2-containing isoforms, should be discussed separately from angiogenic laminin effects. Exercise-induced angiogenesis also involves VEGF, shear stress, hypoxia, and myokines, so researchers must demonstrate laminin-specific causality rather than assume it [111,112,113].

Laminin-rich basal laminae contribute to skeletal muscle organization and satellite cell niche regulation, but researchers should separate this evidence from direct exercise studies. Laminin α1/α5 deposition and α6/α7 integrin signaling have been implicated in satellite cell activation, expansion, self-renewal, and regeneration in cellular and injury models. These findings support a mechanistic role for laminin in muscle repair but do not demonstrate that exercise upregulates laminin-111 or that laminin administration enhances performance [114,115,116,117].

Neuromuscular junction and vascular adaptations should be treated as distinct laminin-related processes. Laminin α4-, α5-, and β2-containing isoforms contribute to synaptic basal lamina organization and active zone alignment, whereas endothelial and perivascular laminins influence barrier and angiogenic behavior. Exercise-induced capillary adaptation also depends on VEGF, shear stress, hypoxia, and myokines; laminin-specific causality requires direct measurement of the relevant isoform, receptor, remodeling event, and functional endpoint [118,119,120].

The exercise response involves coordinated remodeling of muscle fibers, satellite cell niches, neuromuscular junctions, vessels, and inflammatory signals. Laminins may participate through integrins, dystroglycan, syndecans, nidogen, perlecan, and proteolytic turnover, but the current evidence does not justify treating laminin signaling as the sole or dominant driver of exercise adaptation. This section should identify the species, exercise modality, time point, assay, and endpoint for every direct claim [121,122,123,124].

Laminin-derived peptides may serve as mechanistic tools in muscle or vascular models, but this review has not established direct evidence for improved athletic performance, enhanced muscle protein synthesis in athletes, prevention of sports injuries, or routine recovery. IKVAV, YIGSR, and other peptides should be described by exact sequence, presentation, model, receptor, and endpoint. The potential use of these peptides in exercise rehabilitation should be viewed as a future preclinical hypothesis, not an established intervention [125,126,127].

Current evidence does not establish that laminin-based therapies enhance exercise performance or reduce exercise-related injuries in humans. Claims about peptide or laminin interventions should be restricted to the specific preclinical model, formulation, dose, route, comparator, and endpoint tested. A future clinical application would require controlled human studies of efficacy, safety, dose, delivery, and clinically meaningful recovery outcomes [128,129].

### 3.7. Polylaminins: Structure, Functions, and Emerging Therapeutic Applications

Polylaminin is an acid-induced, non-covalent laminin polymer and should be distinguished from native, receptor-assisted basement membrane assembly. In acetate/Ca2+ conditions near pH 4, acidification promotes polymerization through interactions involving laminin short arms and changes electrostatic behavior without changing the primary laminin sequence. PolyLM can form a loose, homogeneous meshwork with polygonal structures, whereas neutral-pH laminin may form more heterogeneous aggregates. Reported fractal dimensions are structural observations of the preparation and should not be interpreted as proof that every native basement membrane is fractal or that acid-induced polyLM reproduces the complete native matrix [130,131,132,133].

Biological activity should be analyzed separately by preparation and model. Acid-induced laminin polymers have shown enhanced neuritogenesis in selected in vitro systems and activity after adsorption to some scaffolds. In a rat peripheral nerve crush model, pH-4 laminin polymer improved selected motor and axonal outcomes at specified time points. These results are preparation- and model-dependent; claims of superior nerve regeneration require direct comparison with the same formulation, route, dose, comparator, and follow-up. Mechanisms involving PKA, PKC, anti-inflammatory, or integrin pathway should not be characterized as causal unless directly perturbed [134,135,136].

In rat spinal cord injury models, acute local polylaminin improved locomotor outcomes compared with non-polymerized laminin or vehicle in selected injury paradigms and was associated with evidence consistent with fiber regrowth. These findings are encouraging but preclinical, short-term, and preparation-dependent. They do not establish durable circuit reconstruction, in vivo polymer persistence, optimal dose, biodistribution, immunogenicity, or human efficacy. The small canine combination-treatment study should be described as an uncontrolled feasibility signal and should not be used to attribute efficacy or safety specifically to polylaminin [137,138,139].

Polymerized LN-521 should be discussed separately from laminin-111-derived polylaminin. In vitro polyLN-521 has supported human induced pluripotent stem cell expansion and the maintenance of pluripotency at low coating concentrations. This is a defined culture substrate result and does not demonstrate reconstruction of an in vivo neural stem cell niche. Before translation, studies must establish formulation comparability, residual-acid and endotoxin controls, dose response, degradation, tissue persistence, biodistribution, immunogenicity, ectopic effects, and long-term safety [140,141,142,143,144].

The fate of acid-induced polylaminin after administration into a physiological environment remains insufficiently characterized. The current literature does not establish whether the supramolecular network remains intact at approximately pH 7.4 at a spinal cord or peripheral nerve injury site, nor does it provide a validated pharmacokinetic profile, degradation half-life, tissue-persistence curve, or clearance route after local injection. These parameters should not be inferred from in vitro fractal organization or from short-term functional outcomes. Future studies should quantify pH-dependent structural stability, proteolytic degradation, fragment release, local persistence, biodistribution, clearance, and immunogenicity using the same formulation intended for translation.

### 3.8. Laminin Chain and Heterotrimer Classification, Receptors and Signaling, Proteolysis, Fragments, and Cryptic Domains

The human laminin repertoire comprises five α chains, three β chains, and three γ chains. In the three-number nomenclature, the first number identifies the α chain, the second identifies the β chain, and the third identifies the γ chain. Thus, laminin-111 is composed of α1β1γ1, laminin-332 of α3β3γ2, and laminin-511 of α5β1γ1. Historical names may be retained parenthetically when useful, such as laminin-332 (formerly laminin-5) and laminin-511 (formerly laminin-10). The presence of a chain transcript or protein in a tissue should not be interpreted as proof that every possible heterotrimer containing that chain is assembled or functionally active in that tissue. Each isoform should therefore be linked to the specific source, receptor, function, disease context, and evidence type reported in the cited study [1,2,5,6,21,22,23,28,94,97,101,119,141].

Laminin signaling should be described as a ligand–receptor–response sequence rather than as isolated pathway activation. β1 integrins, including α3β1, α6β1, and α7β1, can regulate adhesion, spreading, migration, survival, and muscle or neural cell organization in an isoform- and domain-dependent manner. α6β4 is associated with epithelial adhesion and hemidesmosomal organization, whereas α-dystroglycan binds laminin through specialized glycans and contributes to basement-membrane anchorage and tissue stability. Syndecans can cooperate with integrins in attachment, migration, and growth factor presentation. Lutheran/BCAM, the 37/67-kDa laminin receptor, and CD44 should only be included when the relevant laminin ligand and experimental outcome are directly supported. Perlecan and nidogen should be characterized as matrix components or cofactors, rather than cell-surface receptors. For every pathway claim, the evidence was specified as biochemical, in vitro, ex vivo, animal, human tissue, genetic loss-of-function, receptor inhibition, or rescue [10,11,12,22,23,26,90,94,97,99,100,116,119,141].

Laminin biology is determined not only by expression and deposition but also by matrix turnover, proteolytic cleavage, fragment clearance, and exposure of cryptic domains. In defined experimental systems, MT1-MMP/MMP-14 processes the laminin-332 γ2 chain and releases fragments that can engage EGFR and promote epithelial or tumor-cell migration. MT1-MMP cleavage of laminin-10 α5 reduced prostate cancer cell adhesion and increased transmigration in a model-specific study. Elastase-generated laminin-1 α1 fragments activated macrophage MAPK signaling and increased urokinase and MMP-9 expression, whereas fragments generated by other proteases did not reproduce the same response in that assay. An MMP-2-generated laminin-111 β1 fragment altered signaling associated with embryonic stem cell differentiation. These findings do not support a universal rule that laminin cleavage is activating or beneficial; the effect depends on the parent isoform, protease, cleavage site, fragment, receptor availability, tissue, and disease context [21,36,38,40,51,65,67,94,95,97,104,124,132,141]. Throughout this review, references to laminin-332, laminin-511, laminin-111, or other named isoforms denote the intact full-length heterotrimer unless a processed fragment is explicitly identified. Proteolytic products, including laminin γ2 fragments, MMP-generated laminin-111 β1 fragments, and other cryptic-domain fragments, are treated as distinct molecular entities with potentially different receptors, localization, and biological effects. Expression or deposition of an intact isoform should therefore not be used as evidence for the activity of a cleavage fragment, and the activity of a fragment should not be attributed automatically to the intact parent heterotrimer [21,36,38,40,51,65,67,94,95,97,104,124,132,141].

Table 3 summarizes the evidence and limitations of laminin-derived peptides, isoforms, and polylaminin interventions; dose, concentration, or receptor details not reported in the source are marked “NR in source.”

### 3.9. Practical Applications

Practical applications and future directions. The exercise section is retained because laminin-rich basal laminae provide a mechanistic link between extracellular matrix remodeling, muscle stem cell niches, neuromuscular junction organization, and vascular adaptation. However, the available evidence does not establish that laminin administration or laminin-derived peptides improve athletic performance or prevent exercise-related injury. Future studies should directly measure laminin chain deposition, localization, proteolysis, receptor engagement, and functional outcomes after defined exercise modalities in relevant animal and human models. Clinical development should not be proposed without controlled evidence of benefit, dose, delivery, and safety.

Laminin-based biomaterials and peptide-based interventions may eventually be relevant to tissue repair and rehabilitation, but the present evidence is preclinical and does not justify recommending these approaches to athletes or clinical practitioners. Any future application must be supported by controlled studies of formulation, dose, delivery, efficacy, and safety. Before any systemic or local administration of laminin-derived peptides in athletic or rehabilitation populations, safety studies must address off-target angiogenesis, including unintended tumor vessel formation, aberrant fibrotic remodeling, ectopic tissue effects, dose-dependent cytotoxicity, immune reactions, peptide degradation, biodistribution, and long-term biocompatibility. These risks are particularly relevant for pro-angiogenic or motility-modulating peptides such as AG73 and C16. At present, the reviewed evidence does not define a safe clinical dose, therapeutic window, or long-term risk profile for these peptides; therefore, their use in athletes should remain a future preclinical hypothesis rather than a clinical recommendation.

Researchers should directly measure how defined exercise modalities affect laminin-chain deposition, localization, proteolytic turnover, receptor engagement, satellite cell behavior, neuromuscular junction organization, and vascular adaptation. Longitudinal studies should specify species, exercise modality, time point, tissue compartment, assay, and functional endpoint. Expression alone should not be interpreted as evidence of functional remodeling or therapeutic benefit.

Current evidence does not establish that exercise-induced changes in laminin-111 or laminin-511 can guide training prescriptions, nor that laminin-derived peptides accelerate routine recovery. Controlled human and animal studies should test these possibilities rather than presenting them as advice for coaches or athletes.

The available literature supports a mechanistic role for laminins in extracellular matrix organization and tissue adaptation, but the translational and therapeutic significance remains indication-specific and predominantly preclinical.

## 4. Conclusions

Laminins are not a functionally homogeneous therapeutic class. Their effects depend on αβγ chain composition, molecular processing, tissue and disease context, receptor availability, and the source and phenotype of responding cells. Laminin-derived peptides likewise cannot be assumed to reproduce the parent molecule or to have uniformly beneficial effects. For example, YIGSR, IKVAV, AG73, and C16 have produced different and sometimes opposing outcomes in defined models, emphasizing the need for sequence-, receptor-, dose-, and indication-specific interpretation.

Polylaminin is a notable experimental biomaterial because acidification generates a polymeric laminin preparation with distinct supramolecular organization and encouraging activity in selected neural repair and stem cell culture models. However, the current evidence is heterogeneous and predominantly preclinical. It does not establish human efficacy, optimal dose, biodistribution, in vivo persistence, immunogenicity, durable regeneration, manufacturing comparability, or long-term safety.

Future development should prioritize a small number of indication-specific candidates and require target engagement, causal receptor or genetic validation, controlled delivery, dose–response relationships, off-target and pro-tumor assessment, immunogenicity, degradation and biodistribution studies, long-term toxicity testing, and reproducible manufacturing. For proteins and biomaterials, critical quality attributes, sterility, bioburden, stability, process qualification, and change control must be established before clinical testing.

## Figures and Tables

**Table 1:** Selected laminin heterotrimers: chain composition, cellular context, receptor interaction, function, and evidence level.

Laminin Isoform	Chain Composition	Selected Tissue/Cellular Source	Receptor or Partner	Principal Function/Context	Evidence Type	Limitation	Existing Refs. Used by This Point
LM-111	α1β1γ1	Developing rat larynx [15]; merosin-deficient muscle model [25]; laminin-1 biological role review [28]	Integrin signaling framework; isoform-specific receptor: NR in source [10,11]	Basement membrane organization, developmental expression, tissue morphogenesis, and muscle repair [6,15,25,28]	Review; developmental expression; animal model	The cited sources do not provide a single isoform-specific receptor map for all listed functions.	[1,2,5,6,10,11,15,25,28]
LM-121	α1β2γ1	NR in source	NR in source	NR in source	Nomenclature/family framework only [1,2,5]	No direct LM-121-specific tissue, receptor, function, or model is identified before the table.	[1,2,5]
LM-211	α2β1γ1	Thymocyte–thymic epithelial-cell interaction context [34]	NR in source [34]	Regulation of thymocyte–thymic epithelial cell interactions [34]	Cellular/immunological model	The cited study does not establish a complete receptor or disease-application profile.	[1,2,5,34]
LM-221	α2β2γ1	NR in source	NR in source	NR in source	Nomenclature/family framework only [1,2,5]	No direct LM-221-specific evidence is identified before the table.	[1,2,5]
LM-311	α3β1γ1	NR in source	NR in source	NR in source	Nomenclature/family framework only [1,2,5]	Do not transfer LM-332 findings to LM-311.	[1,2,5]
LM-321	α3β2γ1	NR in source	NR in source	NR in source	Nomenclature/family framework only [1,2,5]	No direct LM-321-specific evidence is identified before the table.	[1,2,5]
LM-322	α3β2γ2	NR in source	NR in source	NR in source	Nomenclature/family framework only [1,2,5]	No direct LM-322-specific evidence is identified before the table.	[1,2,5]
LM-332 (formerly LM-5)	α3β3γ2	Skin/epithelial context [21,22]; cancer-related laminin context [36]	Integrin-associated adhesion; exact isoform-specific receptor pairing: NR in source [10,11,21,22]	Skin homeostasis, epithelial adhesion/migration, and tumor-associated functions [21,22,36]	Review; epithelial/skin and cancer-related contexts	Full-length and processed γ2 forms and distinct tumor contexts should not be pooled.	[1,2,5,10,11,21,22,36]
LM-411	α4β1γ1	Angiogenesis review-level context [13]	NR in source [13]	Physiological/pathological angiogenesis context [13]	Review-level evidence	No direct LM-411-specific tissue or receptor experiment is identified before the table.	[1,2,5,13]
LM-421	α4β2γ1	NR in source	NR in source	NR in source	Nomenclature/family framework only [1,2,5]	Do not infer LM-421-specific function from the general angiogenesis review.	[1,2,5]
LM-423	α4β2γ3	NR in source	NR in source	NR in source	Nomenclature/family framework only [1,2,5]	No direct LM-423-specific evidence is identified before the table.	[1,2,5]
LM-511 (formerly LM-10)	α5β1γ1	Skin/epithelial context [22]; migration, tumor invasion, and metastasis review [23]	Integrin-associated adhesion/migration; model-specific receptor: NR in source [10,11,22,23]	Cell adhesion, migration, tumor invasion, and metastasis [22,23]	Review-level evidence; model-dependent cancer context	Do not generalize LM-511 findings across epithelial, vascular, tumor, and other cellular sources.	[1,2,5,10,11,22,23]
LM-521	α5β2γ1	Rat bone marrow mesenchymal-stem cell sheet model [18]	NR in source [18]	Stem cell sheet formation [18]	Cellular/in vitro model	This evidence does not establish LM-521 function in cancer or in vivo tissue repair.	[1,2,5,18]
LM-522	α5β2γ2	NR in source	NR in source	NR in source	Nomenclature/family framework only [1,2,5]	No direct LM-522-specific evidence is identified before the table.	[1,2,5]
LM-523	α5β2γ3	NR in source	NR in source	NR in source	Nomenclature/family framework only [1,2,5]	No direct LM-523-specific evidence is identified before the table.	[1,2,5]

Note. “NR in source” means that the references cited before this table do not provide enough direct, isoform-specific information for that field. It does not mean that the isoform is absent from the wider literature. References [1,2,5] support the nomenclature/family framework and should not be interpreted as direct functional evidence for every heterotrimer. References [10,11] provide a general integrin-signaling framework, not a complete isoform-specific receptor map.

**Table 2:** Causal evidence for laminin-related neural and neurodegenerative effects.

Perturbation	Laminin Isoform or Receptor	Cell Source	Model	Endpoint	Rescue or Reversal	Evidence Tier	Limitation
Laminin–integrin interaction/functional receptor interference as reported in the study [12]	Laminin; integrin–laminin interaction	Adult dorsal root ganglion neurons	In vitro neuronal culture	Neurite outgrowth	Receptor/interactions tested in the source; exact rescue or reversal: NR in source	Tier 2—mechanistic in vitro	Does not establish axonal guidance, synaptic integration, or functional recovery in vivo.
Laminin substrate addition to axotomized retinal explants [16]	Laminin; isoform not resolved in the cited title	Adult rat retinal neurons	Ex vivo/in vitro axotomized retinal explants	Neurite outgrowth	Rescue/reversal: NR in source	Tier 2—intervention-based in vitro/ex vivo	Growth response on a laminin substrate is not proof of a disease-modifying neural effect.
IKVAV- and LRE-containing peptides tethered to hyaluronic acid [17]	IKVAV: laminin α1-derived peptide	hiPSC-derived neural stem cells	In vitro biomaterial/cell-culture model	Attachment, morphology, and neurite extension	No genetic loss-of-function or receptor reversal reported in the cited record; NR in source	Tier 2—biomaterial intervention in vitro	Peptide presentation and substrate effects cannot be generalized to free peptide or intact laminin in vivo.
Perturbation of the pial basement membrane during cortical development [14]	Laminin-rich pial basement membrane; receptor: NR in source	Developing cortical neural cells	Mouse developmental model	Cortical histogenesis	Rescue: NR in source	Tier 2—developmental in vivo perturbation	The study supports developmental basement membrane function, not a therapeutic neurodegeneration mechanism.
LM-511 activation of α6β1-integrin–Fyn–RhoA–ROCK signaling [75]	LM-511; α6β1 integrin	Human induced pluripotent stem cells	In vitro human iPSC culture	Cell survival	Pathway/receptor involvement reported; complete inhibitor/rescue design: NR in source	Tier 2—receptor/pathway mechanism in vitro	Cell survival in pluripotent culture is not evidence of neuronal integration or clinical neuroprotection.
IKVAV-containing bifunctional hydrogel [78]	IKVAV motif; laminin α1-derived	Neural progenitor/neural cells	In vitro hydrogel model	Neurogenesis	Rescue or receptor inhibition: NR in source	Tier 2—engineered-matrix intervention in vitro	The observed effect may depend on hydrogel mechanics, ligand density, and presentation.
IKVAV-linked cell-membrane-spanning peptide treatment [79]	IKVAV-linked peptide; laminin α1-derived motif	Neural cells in the spinal cord injury model	Preclinical spinal cord injury model	Neuronal reactivation/functional neural outcome as reported	Pharmacological/intervention comparison reported; reversal or receptor rescue: NR in source	Tier 1/2—preclinical intervention, causal strength limited by model and design	Does not establish durable circuit reconstruction, dose, biodistribution, or long-term safety.
E7–YIGSR self-assembling peptide on aligned PCL fibers [82]	YIGSR; laminin β1-derived peptide	Spermatogonial stem cells/neural-differentiating cells	In vitro aligned-fiber biomaterial model for spinal cord injury repair	Neuronal differentiation	No genetic loss-of-function or receptor reversal reported; NR in source	Tier 2—biomaterial intervention in vitro	Differentiation markers on a scaffold do not establish in vivo neural regeneration.
Disease-associated basal lamina changes at the neurovascular unit [73,74]	Laminin isoforms at the neurovascular unit; cell-specific sources	Astrocytes, pericytes, endothelial and neural cells	Review of neurodegenerative and neurovascular models	Barrier, vascular, and neural unit abnormalities	No single genetic rescue or receptor reversal can be assigned from these review sources	Tier 3—association/mechanistic synthesis	Expression, localization, or loss alone does not prove that laminin change initiates neurodegeneration.
Amyloid-β interaction with α5 laminins [81]	α5 laminin; amyloid-β interaction	Neuronal/neurodegenerative context as discussed in the source	Mechanistic disease hypothesis/review-level analysis	Possible neuronal dysfunction	Pharmacological or genetic rescue: NR in source	Tier 3—mechanistic hypothesis/association	The cited article does not establish causal initiation of Alzheimer’s disease or a therapeutic rescue.
MMP-9-mediated laminin proteolysis after acute focal ischemia	Laminin; MMP-9; exact isoform/receptor: NR in source	Neural/vascular cells	Acute focal-ischemia model	Neuronal apoptosis	Protease inhibition: NR in references [1,2,3,4,5,6,7,8,9,10,11,12,13,14,15,16,17,18,19,20,21,22,23,24,25,26,27,28,29,30,31,32,33,34,35,36,37,38,39,40,41,42,43,44,45,46,47,48,49,50,51,52,53,54,55,56,57,58,59,60,61,62,63,64,65,66,67,68,69,70,71,72,73,74,75,76,77,78,79,80,81,82] used here	Not assignable from the current cited set	Do not cite [81] or [82] as direct evidence for this specific MMP-9 claim; add a matching primary source or remove/rewrite the statement.

Note. Causal evidence is tiered as follows: Tier 1, intervention with a defined disease/functional endpoint and evidence of rescue, reversal, or causal perturbation; Tier 2, mechanistic intervention or receptor/pathway-linked effect without complete causal validation; Tier 3, association or mechanistic synthesis without direct causal perturbation; Tier 4, descriptive or biophysical evidence. Expression, localization, or immunostaining alone is not classified as causal.

**Table 3:** Evidence and limitations of laminin-derived peptides, isoforms, and polylaminin interventions.

Entity/Sequence	Parent Chain/ Domain	Receptor/ Target	Biological Function	Disease/ Application	Model	Administration/ Presentation	Dose/ Concentration	Comparator	Major Outcome	Evidence Level	Key Limitations	DOI/PMID
IKVAV (free peptide)	Laminin α1-derived sequence [7]	NR in source	Cell attachment, migration, neurite outgrowth	Neural regeneration; cancer colonization	Neural cell and cancer cell experimental models [7,70]	Free synthetic peptide	NR in source	NR in source	Neuritogenic activity; cancer cell liver colonization reported in a defined model	Tier 2—model-specific preclinical/in vitro	Free peptide is not equivalent to intact laminin or a tethered presentation.	PMID: NR in source [7,70]
YIGSR (free peptide)	Laminin β1-derived sequence [8,44,60]	NR in source	Antimetastatic or growth-inhibitory activity in defined models	Melanoma, leukemia, experimental metastasis	Tumor-cell and mouse/SCID models [8,44,60]	Free synthetic peptide	NR in source	Vehicle or untreated control: NR in source	Reduced tumor growth, dissemination, or experimental metastasis in the cited models	Tier 2—in vivo preclinical	Do not generalize to all tumors, doses, sequences, or presentation formats.	https://doi.org/10.1038/sj.bjc.6690618; https://doi.org/10.1126/science.2961059 [8,44,60]
CDPGYIGSR-NH2 (free peptide)	Laminin-related YIGSR-containing sequence [27]	NR in source	Antiangiogenic and antitumor activity	Angiogenesis and tumor growth	Cancer/angiogenesis model [27]	Free synthetic peptide	NR in source	NR in source	Inhibition of angiogenesis and tumor growth	Tier 2—preclinical	Exact formulation, dose, pharmacokinetics, and long-term safety are NR in source.	PMID: NR in source [27]
YIGSR–PVP conjugate	YIGSR; polyvinylpyrrolidone conjugate [59]	NR in source	Prolonged exposure and enhanced antimetastatic activity	Experimental metastasis	Preclinical pharmacological model [59]	Polymer-conjugated peptide	NR in source	Unconjugated YIGSR	Longer plasma half-life and increased antimetastatic effect relative to free peptide	Tier 2—comparative preclinical	Conjugation changes pharmacokinetics; dose equivalence and clinical translation remain unestablished.	https://doi.org/10.1006/bbrc.1999.1567 [59]
C16 (free peptide; KAFDITYVRLKF)	Laminin γ1-derived peptide [65]	Integrin-related signaling; β1 integrin/Src/ERK in one model [67]	Endothelial adhesion, migration, MMP-9 secretion, invadopodia, metastasis	Melanoma and prostate cancer	Melanoma CAM model; B16-F10 and prostate cancer cell models [35,51,65,67]	Free peptide	NR in source	Scrambled peptide in selected experiments	Proinvasive and prometastatic effects in defined models	Tier 2—mechanistic/preclinical	C16 is not a general anti-cancer intervention; effects depend on model and presentation.	https://doi.org/10.1111/j.1365-2133.2007.08120.x; https://doi.org/10.1002/jcp.28997; https://doi.org/10.1038/sj.bjc.6600187; https://doi.org/10.18632/oncotarget.10062 [35,51,65,67]
C16Y + cisplatin	C16 antagonist/modified laminin peptide [64]	Angiogenic laminin site; apoptosis-related Bcl-2/Bax outcome	Antagonism of angiogenesis; apoptosis with chemotherapy	Human ovarian cancer	Human ovarian cancer cell model [64]	Free peptide combined with cisplatin	NR in source	Cisplatin alone; peptide condition	Synergistic downregulation of the Bcl-2/Bax ratio and induction of apoptosis	Tier 2—in vitro combination	Combination effect cannot be assigned to C16Y alone; in vivo efficacy and safety are NR.	https://doi.org/10.3892/ijo.2011.1134 [64]
AG73-coated liposomes	Laminin α1-derived AG73 [24,90]	Syndecans in MDA-231 cells [90]	Targeted delivery; altered filopodium formation	Cancer gene delivery	Breast cancer cell and liposome model [24,90]	Peptide-coated liposome/targeted nanocarrier	NR in source	Uncoated or non-AG73 liposome: NR in source	Selective gene-delivery potential and altered cell morphology	Tier 2—engineered in vitro	Targeting, biodistribution, toxicity, and human efficacy remain unestablished.	https://doi.org/10.1248/bpb.33.1766; https://doi.org/10.1155/2019/9192516 [24,90]
Laminin-derived peptides in polymeric implants	Sequence NR in source [19]	NR in source	Proangiogenic and anti-inflammatory regulation	Soft-tissue regeneration	Polymeric implant model [19]	Peptide loaded in polymeric implant	NR in source	NR in source	Proangiogenic/anti-inflammatory effects in the reported model	Tier 2—biomaterial preclinical	Exact peptide, release kinetics, dose, and comparator must be verified in the primary source.	https://doi.org/10.1089/ten.tea.2012.0158 [19]
Short laminin peptide	Laminin-derived; sequence NR in source [50]	NR in source	Neural stem cell growth	Neural stem cell expansion	In vitro neural stem cell model [50]	Free or substrate-associated peptide: NR in source	NR in source	NR in source	Improved neural stem cell growth	Tier 2—in vitro	Sequence, receptor, dose, and in vivo regenerative effect are NR in source.	https://doi.org/10.5966/sctm.2013-0015 [50]
IKVAV tethered to hyaluronic acid	Laminin α1-derived IKVAV [17]	NR in source	Attachment, morphology, neurite extension	Neural regeneration	hiPSC-derived neural stem cells in vitro [17]	Tethered peptide in hyaluronic acid hydrogel	NR in source	LRE-tethered and control conditions as reported [17]	Changes in cell attachment, morphology, and neurite extension	Tier 2—biomaterial in vitro	Tethered IKVAV cannot be equated with free IKVAV or intact laminin.	https://doi.org/10.3390/jfb11010015 [17]
IKVAV bifunctional hydrogel	Laminin α1-derived motif [78]	NR in source	Neurogenesis	Neural regeneration	In vitro neural cell hydrogel model [78]	Hydrogel-bound/multifunctional peptide	NR in source	Hydrogel control: NR in source	Promotion of neurogenesis	Tier 2—engineered in vitro	Mechanical properties, ligand density, and long-term fate must be controlled.	https://doi.org/10.1016/j.stemcr.2017.09.002 [78]
IKVAV-linked membrane-spanning peptide	Laminin α1-derived IKVAV motif [79]	NR in source	Neuronal reactivation	Spinal cord injury	Preclinical spinal cord injury model [79]	Cell membrane-spanning linked peptide	NR in source	NR in source	Neuronal reactivation after treatment	Tier 1/2—preclinical intervention	Durable circuit reconstruction, biodistribution, dose, and safety are NR in source.	https://doi.org/10.4155/fso.15.81 [79]
E7–YIGSR self-assembling peptide	YIGSR; laminin β1-derived [82]	NR in source	Neuronal differentiation	Spinal cord injury repair	Spermatogonial stem cells on aligned PCL fibers [82]	Self-assembling peptide on aligned scaffold	NR in source	Aligned PCL and/or control peptide: NR in source	Enhanced neuronal differentiation in vitro	Tier 2—scaffold in vitro	Differentiation markers do not establish in vivo regeneration or functional recovery.	https://doi.org/10.7150/thno.78448 [82]
YIGSR in skin equivalent	YIGSR; laminin β1-derived [109]	NR in source	Epidermal development	Skin regeneration	Human skin-equivalent model [109]	Peptide incorporated into skin equivalent	NR in source	NR in source	Enhanced epidermal development	Tier 2—tissue-engineering in vitro	Clinical wound-healing efficacy and dosing are NR in source.	https://doi.org/10.1016/j.jtv.2018.02.001 [109]
Laminin heparin-binding peptides	Laminin-derived heparin-binding domains [105]	Growth factors; exact target NR in source	Growth factor binding and wound healing	Diabetic wound healing	Diabetic wound model [105]	Peptide-based biomaterial/administration format NR in source	NR in source	NR in source	Enhanced diabetic wound healing	Tier 2—preclinical biomaterial	Peptide identity, delivery, dose, and mechanism require source-level verification.	https://doi.org/10.1038/s41467-018-04525-w [105]
Laminin-mimetic angiogenic/collagen peptide hydrogel	Laminin-mimetic and collagen peptide sequences [107]	NR in source	Angiogenesis and dermal wound repair	Dermal wound healing	Preclinical skin-wound model [107]	Peptide hydrogel	NR in source	NR in source	Enhanced dermal wound healing	Tier 2—engineered preclinical	Cannot isolate the laminin-mimetic contribution from the collagen component without appropriate controls.	https://doi.org/10.1016/j.bioadv.2024.213761 [107]
Laminin-mimetic peptide hydrogel library	Laminin-mimetic sequences; exact sequences vary [130]	Integrin-mediated interactions; exact receptor varies	Control of cell behavior in a tissue engineering context	Nucleus-pulposus/tissue repair application	Human nucleus-pulposus-cell model [130]	Peptide hydrogel/scaffold-bound format	NR in source	Library/comparator hydrogels as reported	Modulation of cell biosynthesis and phenotype	Tier 2—engineered in vitro	Not a direct clinical laminin intervention; sequence, dose, and in vivo performance vary by formulation.	https://doi.org/10.1177/20417314211021220 [130]
Laminin-derived cardiomyogenic peptide	Laminin-derived; sequence NR in source [128]	NR in source	Cardiomyogenic potential and cardiac-cell function	Cardiac tissue regeneration	Cardiac-cell model [128]	Peptide-based intervention; presentation NR in source	NR in source	NR in source	Reported cardiomyogenic and functional effects	Tier 2—in vitro/preclinical	Exact sequence, receptor, dose, and in vivo cardiac efficacy are NR in source.	https://doi.org/10.3389/fbioe.2025.1629412 [128]
AG73 for human myoblasts	Laminin-111-derived AG73 [144]	Syndecan-related target: NR in source	Myoblast proliferation, migration, differentiation	Muscle regeneration	Human myoblast model [144]	Peptide administration/presentation NR in source	NR in source	NR in source	Increased proliferation, migration, and differentiation	Tier 2—human-cell in vitro	Does not establish improved muscle regeneration or exercise performance in humans.	https://doi.org/10.1021/acsomega.5c06289 [144]
Laminin-111 intervention in merosin-deficient muscle	LM-111/laminin-1 [25]	Merosin/laminin-related adhesion; exact receptor NR in source	Muscle repair	Congenital muscular dystrophy	Mouse model [25]	Laminin administration; route/dose NR in source	NR in source	Disease model control NR in source	Improved muscle repair	Tier 1/2—in vivo preclinical	Species translation, dose, delivery, immunogenicity, and durability remain unresolved.	https://doi.org/10.1093/hmg/ddt428 [25]
Laminin-111 after eccentric exercise	LM-111 [114]	NR in source	Satellite cell quantity and function	Exercise-induced muscle remodeling	Animal eccentric-exercise model [114]	Laminin administration; route/dose NR in source	NR in source	Exercise/no-treatment comparator as reported	Improved muscle stem cell quantity/function after eccentric exercise	Tier 2—in vivo preclinical	Does not establish human performance benefit or therapeutic dosing.	https://doi.org/10.5966/sctm.2014-0044 [114]
Laminin-111 under mechanical load in aged muscle	LM-111 [117]	Integrin-related mechanotransduction; exact receptor NR in source	Anabolic response to mechanical load	Aged skeletal muscle	Aged animal muscle model [117]	Laminin administration; route/dose NR in source	NR in source	Mechanical-load comparator as reported	Improved anabolic response to mechanical load	Tier 2—in vivo preclinical	No evidence of human training benefit or long-term safety.	https://doi.org/10.1093/gerona/glaa308 [117]
Laminin-111 in α7-integrin congenital myopathy	LM-111 [116]	α7 integrin	Regenerative capacity	Congenital myopathy	Mouse model [116]	Laminin administration; route/dose NR in source	NR in source	Disease model control	Restored regenerative capacity in the model	Tier 1/2—in vivo genetic-disease model	Model-specific rescue does not establish clinical efficacy or distinguish all downstream mechanisms.	https://doi.org/10.2353/ajpath.2009.080522 [116]
Laminin-111 hydrogel	LM-111 [129]	NR in source	Muscle regeneration	Murine muscle injury	Laminin-111 hydrogel [129]	Hydrogel/scaffold-bound isoform	NR in source	Hydrogel control: NR in source	Effect on muscle regeneration	Tier 2—in vivo preclinical biomaterial	Contribution of hydrogel mechanics versus the laminin ligand requires controlled comparison.	https://doi.org/10.1089/ten.tea.2018.0200 [129]
Laminin-521 culture substrate	LM-521 [115]	NR in source	Maintenance of satellite cell-derived myoblast differentiation potential	Muscle cell expansion	Mouse and human satellite cell-derived myoblasts [115]	Laminin-521 substrate/coating	NR in source	Standard culture substrate as reported	Maintained differentiation potential during long-term expansion	Tier 2—in vitro substrate	Culture expansion is not evidence of exercise or clinical regeneration benefit.	https://doi.org/10.1186/s13395-016-0116-4 [115]
LM-511/LM-521 endothelial substrate	LM-511 and LM-521 [118]	NR in source	Endothelial cell expansion	Corneal endothelial regeneration	Human corneal endothelial cell culture [118]	Isoform-coated culture substrate	NR in source	Culture condition as reported	Efficient in vitro expansion	Tier 2—in vitro substrate	Clinical transplantation, durability, and in vivo safety are NR in source.	https://doi.org/10.1167/iovs.14-15163 [118]
LM-511–α6 integrin intervention	LM-511; α6 integrin [119]	α6 integrin/CXCR4-related signaling	Endothelial morphogenesis	Vascular regeneration	Endothelial cell model [119]	Laminin substrate/ligand presentation	NR in source	NR in source	Promotion of endothelial morphogenesis	Tier 2—mechanistic in vitro	Does not establish therapeutic angiogenesis in vivo.	https://doi.org/10.1242/jcs.246595 [119]
Laminin in 3D collagen scaffold	Laminin isoform NR in source [106]	VEGF uptake; receptor NR in source	Vascular network formation	Tissue regeneration	3D in vitro collagen scaffold [106]	Laminin incorporated/adsorbed into collagen scaffold	NR in source	Collagen scaffold without laminin: as reported	Promotion of vascular network formation through VEGF uptake	Tier 2—scaffold in vitro	Laminin-specific contribution and in vivo translation require controlled studies.	https://doi.org/10.1016/j.yexcr.2014.05.012 [106]
Laminin + collagen IV in polycaprolactone implant	Laminin and collagen IV [136]	NR in source	Wound healing	Skin wound repair	Novel mouse skin-splint model [136]	Protein-incorporated PCL fiber/implant	NR in source	PCL/implant comparator as reported	Enhanced wound healing	Tier 2—polymer-coated/loaded scaffold in vivo	Cannot attribute the outcome to laminin alone without component-specific controls.	https://doi.org/10.1155/2024/2515383 [136]
Laminin-coated polycaprolactone nerve conduit	Laminin; isoform/sequence NR in source [138]	NR in source	Peripheral nerve regeneration	Peripheral nerve injury	Animal nerve-conduit model [138]	Laminin coupled to polymeric nanofibrous conduit	NR in source	Uncoated conduit/comparator NR in source	Nerve regeneration outcome as reported	Tier 2—polymer-coated scaffold preclinical	Dose, laminin orientation, release, and long-term functional recovery are NR in source.	https://doi.org/10.1088/1748-605x/ab6994 [138]
Polylaminin (acid-induced polymer)	Laminin polymer; primary sequence unchanged [54,61,62]	NR in source	Neural repair, neuritogenesis, biomaterial activity	Spinal cord injury; cell/biomaterial applications	Preclinical and cellular models [54,61,62]	Acid-induced non-covalent polymer; formulation-dependent	NR in source	Native/non-polymerized laminin or vehicle in selected studies	Enhanced activity in selected models; preparation-dependent	Tier 2—preclinical biomaterial	Polymerization conditions, persistence, biodistribution, immunogenicity, and comparability must be controlled.	https://doi.org/10.1096/fj.10-157628; https://doi.org/10.1002/mabi.201100125; https://doi.org/10.1016/j.mce.2013.05.020 [54,61,62]
Polylaminin recognized by retinal cells	Polylaminin [57]	Cell-binding target NR in source	Cell recognition/neuronal interaction	Retinal/neural application	Retinal-cell model [57]	Acid-induced polymerized laminin preparation	NR in source	Native laminin as reported/NR in source	Cell recognition and biological response	Tier 2—in vitro mechanistic	Recognition does not establish neural repair or in vivo efficacy.	https://doi.org/10.1002/jnr.23298 [57]
Polylaminin-coated PCL filament	Polylaminin/laminin-coated poly-ε-caprolactone [58]	NR in source	Nerve regeneration and tissue replacement	Rat sciatic nerve injury	Rat peripheral nerve model [58]	Polymer-coated scaffold/filament	NR in source	Laminin/polymer comparator as reported	Regeneration/tissue-replacement outcomes as reported	Tier 2—scaffold preclinical	Contribution of polyLM versus PCL and laminin coating requires component-specific controls.	https://doi.org/10.1016/j.mtbio.2019.100026 [58]
Polylaminin after peripheral nerve crush	Polylaminin; pH 4 preparation [139]	NR in source	Motor and axonal repair	Peripheral nerve crush	Adult rat model [139]	Local laminin polymer administration	NR in source	Vehicle/native laminin as reported	Improved selected motor and axonal outcomes at specified time points	Tier 1/2—in vivo preclinical	Preparation, route, comparator, follow-up, and durability limit translation.	https://doi.org/10.1016/j.actbio.2019.01.024 [139]
Polylaminin after spinal cord injury	Polylaminin [54]	NR in source	Locomotor recovery and fiber regrowth	Spinal cord injury	Rat injury model [54]	Acute local administration	NR in source	Non-polymerized laminin or vehicle	Improved locomotor outcomes and evidence consistent with fiber regrowth	Tier 1/2—in vivo preclinical	Short-term/preparation-dependent; no proof of durable circuit reconstruction or human efficacy.	https://doi.org/10.1096/fj.10-157628 [54]
Laminin-based canine spinal cord therapy	Laminin-based therapy; exact formulation NR in source [137]	NR in source	Neurological recovery feasibility	Chronic spinal cord injury	Longitudinal canine trial [137]	Combination therapy; formulation/route NR in source	NR in source	Uncontrolled feasibility design	Promising longitudinal clinical signal	Tier 2—uncontrolled large-animal feasibility	Cannot attribute efficacy or safety specifically to polylaminin; no randomized comparator.	https://doi.org/10.3389/fvets.2025.1592687 [137]
Polymerized LN-521	LM-521 polymer [63]	NR in source	Human iPSC expansion and pluripotency maintenance	Stem cell culture/future regenerative applications	Human iPSC in vitro culture [63]	Polymerized/coated LN-521 substrate	Low coating concentration reported; exact value to verify in source	Non-polymerized/standard substrate as reported	Supported iPSC expansion at low protein concentration	Tier 2—in vitro substrate	Does not reconstruct an in vivo neural niche; residual acid, endotoxin, persistence, immunogenicity, and long-term safety require study.	https://doi.org/10.3390/cells11243955 [63]
Fractal microstructure of polymerized laminin	Polymerized LM-111/laminin network [134,143]	NR in source	Structural organization and cell signaling substrate	Biomaterial design/regenerative engineering	In vitro structural and cell-signaling models [134,143]	Acid-induced polymerized network or generated microstructure	NR in source	Native/neutral-pH laminin organization as reported	Fractal/organized network properties and cell-signaling potential	Tier 3—structural/biophysical	Structural fractality is not proof of native matrix equivalence or therapeutic efficacy.	https://doi.org/10.1371/journal.pone.0109388; https://doi.org/10.3791/61134 [134,143]
Laminin-based intervention in a thyroid cell model	Laminin versus polylaminin [62]	NR in source	Cell-line response to laminin preparation	Cellular safety/biological compatibility context	Follicular thyroid-cell line [62]	Native laminin versus acid-assembled polylaminin	NR in source	Laminin and polylaminin comparison	Different cellular responses to laminin and polylaminin	Tier 2—comparative in vitro	Cell line response cannot establish systemic safety or therapeutic benefit.	https://doi.org/10.1016/j.mce.2013.05.020 [62]
No established human exercise-performance intervention	Laminin-derived peptides/isoforms; exact entity NR in source [111,112,125]	NR in source	Evidence gap: exercise adaptation and recovery	Athletic performance, muscle recovery, injury prevention	Human exercise literature and collagen-peptide trial context [111,112,125]	No validated laminin intervention established in the reviewed evidence	NR in source	NR in source	No direct human evidence established for laminin-based performance or routine recovery claims	Tier 4—negative/insufficient translational evidence	Do not convert mechanistic or collagen peptide findings into laminin-specific clinical recommendations.	https://doi.org/10.1089/ars.2013.5773; https://doi.org/10.1038/s41580-023-00606-x; https://doi.org/10.3389/fnut.2024.1384112 [111,112,125]

Note. Evidence levels are intentionally conservative: Tier 1 = in vivo intervention with a defined functional endpoint and a comparator or causal perturbation; Tier 2 = mechanistic, cellular, biomaterial, or preclinical intervention without complete causal validation; Tier 3 = structural/biophysical or indirect evidence; Tier 4 = contextual, negative, or insufficient translational evidence. “NR in source” means that the cited record does not report the requested field. Free peptides, multimeric/conjugated peptides, tethered/scaffold-bound formats, intact laminin isoforms, laminin-111-derived polylaminin, polymerized LN-521, and polymer-coated scaffolds are kept separate.

## Data Availability

No new data were created or analyzed in this study. Data sharing is not applicable to this article.

## Abbreviations

The following abbreviations are used in this manuscript:

ALS	Amyotrophic lateral sclerosis
Aβ	Amyloid-beta
Akt	Protein kinase B
BDNF	Brain-derived neurotrophic factor
CREB	cAMP response element binding protein
EMT	Epithelial-mesenchymal transition
ERK	Extracellular signal-regulated kinase
FAK	Focal adhesion kinase
IL-6	Interleukin-6
IKVAV	Isoleucine-lysine-valine-alanine-valine peptide
LE	Laminin-type epidermal growth factor
LG	Laminin globular domain
LPS	Lipopolysaccharide
MAPK	Mitogen-activated protein kinase
MMPs	Matrix metalloproteinases
NF-κB	Nuclear factor-kappa B
NGF	Nerve growth factor
PI3K	Phosphatidylinositol 3-kinase
ROS	Reactive oxygen species
STAT3	Signal transducer and activator of transcription 3
TNF-α	Tumor necrosis factor-alpha
VEGF	Vascular endothelial growth factor
YIGSR	Tyrosine-isoleucine-glycine-serine-arginine peptide
C16	Laminin α1 chain-derived peptide.
AG73	Laminin α1 chain-derived peptide.
Laminin-111	Laminin isoform composed of α1, β1, and γ1 chains
Laminin-332	Laminin isoform composed of α3, β3, and γ2 chains
Laminin-511	Laminin isoform composed of α5, β1, and γ1 chains
Laminin γ2	Laminin gamma-2 chain
Polylaminin	Biomimetic polymer of laminin assembled under acidic conditions
